# Identification of the molecular relationship between intravenous leiomyomatosis and uterine myoma using RNA sequencing

**DOI:** 10.1038/s41598-018-37452-3

**Published:** 2019-02-05

**Authors:** Xu Zhang, Liangcai Wu, Rongjian Xu, Chengpei Zhu, Guotao Ma, Chaoji Zhang, Xingrong Liu, Haitao Zhao, Qi Miao

**Affiliations:** Chinese Academy of Medical Sciences, Peking Union Medical College Hospital, Beijing, China

## Abstract

The purpose of this study was to explore the potential relationship between intravenous leiomyomatosis (IVL) and uterine myoma (UM) at the molecular level. RNA-sequencing was performed on IVL tumours, UM tumours, and adjacent normal uterine muscle. We compared the gene expression levels between IVL and normal uterine muscle, UM and normal uterine muscle, to identify differentially expressed genes (DEGs). Then we used Gene Ontology Enrichment Analysis to determine the functions of the DEGs and performed specimen cluster analysis. We obtained 98 DEGs between IVL and adjacent normal uterine muscle, and 61 DEGs between UM and adjacent normal uterine muscle. Functional enrichment of both IVL and UM DEGs showed that they are associated with hormone stimulus, extracellular matrix, and cell adhesion. Unsupervised clustering analysis showed that IVL and UM could not be separated completely. Among these dysregulated genes, we found that HOXA13 showed a distinct dysregulated status between IVL and UM. HOXA13 may therefore serves as a biomarker to distinguish IVL and UM. Our results showed that IVL and UM may have similar dysregulated gene networks. They may be closely related, and HOXA13 may serves as a biomarker to distinguish between IVL and UM.

## Introduction

**Figure 1:**
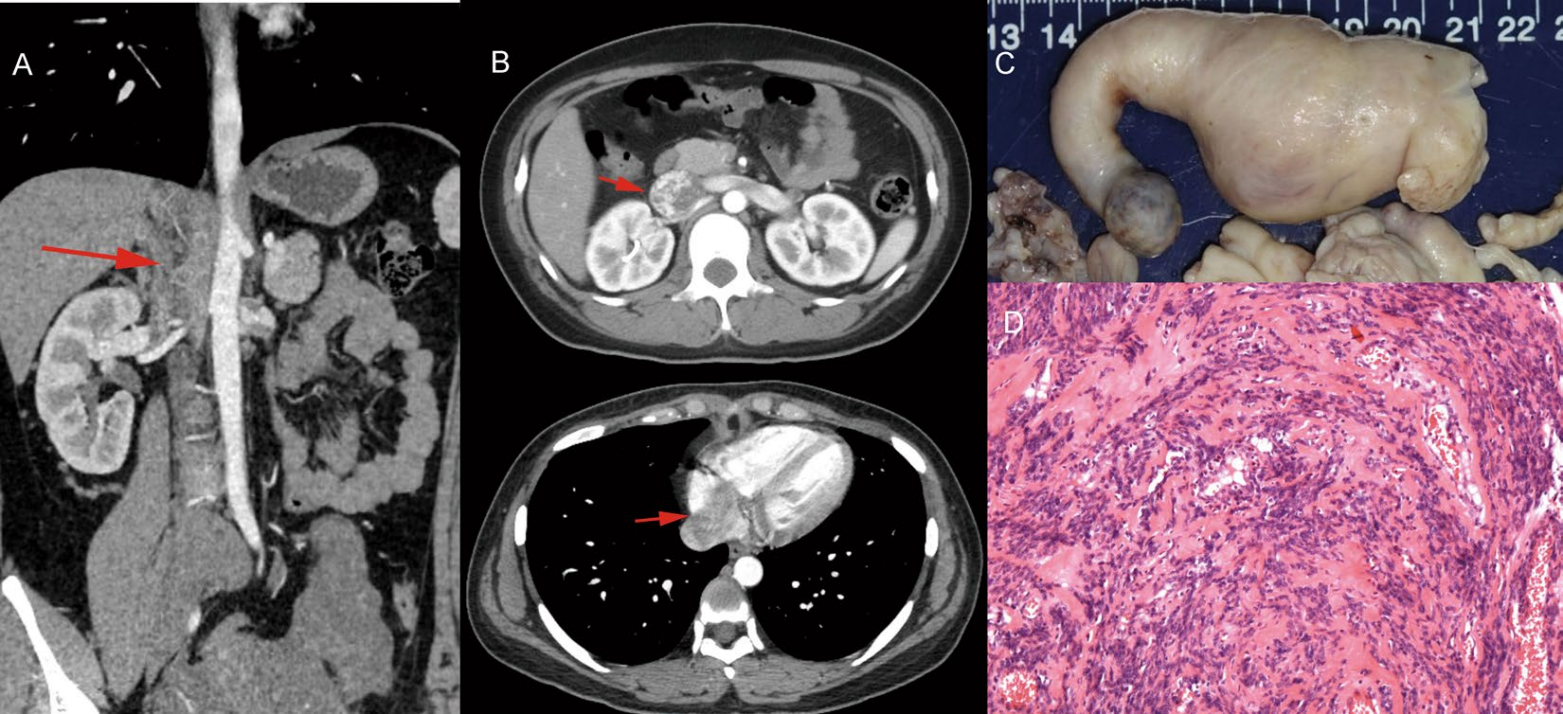
(**A**,**B**) IVL (red arrow) originated from the Iliac vein, filled in the Inferior vena cava and extended into the right heart atrium. (**C**) The morphology of IVL which was excised from Inferior vena cava and right heart atrium. (**D**) HE staining of IVL indicated that it is similar to the benign leiomyoma.

Intravenous leiomyomatosis (IVL) is a rare benign tumour of pelvic origin. It was first described by Hirschfield in 1896^1^. To date, more than 200 cases have been reported in English literature worldwide. With accumulating knowledge of IVL, many of its features have been characterized. It occurs among women patients with the median age of 45 years old. Symptoms include edema of lower extremity, dyspnea, fatigue, tachycardia, abdominal discomfort and may even result in sudden death^2^. Typically, IVL is regarded as a benign vascular tumour consisting of vascular smooth muscle cells. Biologically, it is characterized as a malignant lesion because it can extend into heart^3^ or metastasize to the lungs^4,5^ (Fig. 1), leading to death in certain cases. The only known curative treatment is surgery^6^.

Uterine myoma is one of the most common benign tumours in women^7^. Several subtypes have been identified, including plexiform leiomyoma and benign metastasizing leiomyoma^8^. Between November 2002 and January 2015, there were 30,757 patients diagnosed with uterine myoma and who received treatment at Peking Union Medical College Hospital. Of those, 76 (0.25%) were also diagnosed with IVL^9^. Between November 2002 and June 2016, we performed surgery on 41 IVL patients, of which 30 (73.2%) had previously undergone uterine myoma surgery (Table 1). These information suggested that there might be a relationship between IVL and uterine myoma; although no definitive conclusions have yet been drawn on this issue.

**Table 1:** The basic information of the 41 patients who receive the surgery in PUMCH cardiac surgery department from 2002 to 2016.

**Summary of patient (n = 41)**	
Presentation	Number (%)
Total cases	n = 41
Age (year)	44.6 ± 6.2
Previous surgery history	
Cardiac tumor resection	2 (4.9%)
hysterectomy/myomectomy	30 (73.2%)
Origin of tumor	
from the genital vein	15 (36.7%)
from the iliac vein	19 (46.3%)
from the iliac vein and the genital vein	6 (14.6%)
Clinical manifestations	
Dyspnea, Chest tightness, shortness of breath	22 (53.7%)
Double lower limb edema	13 (31.7%)
Syncope	4 (9.8%)
Serous cavity effusion	4 (9.8%)
Pelvic mass	32 (78%)
Pulmonary embolism	4 (9.8%)
Pulmonary metastasis	3 (7.3%)
Lack of symptom	10 (24.4%)

There are two prevailing hypotheses about the pathogenic relationship between IVL and uterine myoma. One theory suggests that IVL is derived from smooth muscle cells in the vessel wall^10^. The other suggests that IVL is derived from uterine myoma^11,12^. However, owing to technological limitations, few reported studies explored the underlying relationship between them. In this study, we used RNA sequencing^13^ to shed light on the molecular relationship between IVL and uterine myoma.

## Results

### Heterogeneous gene expression in IVL

The morphology of IVL is often irregular. In addition, a common phenomenon of IVL is intracardiac extension with the potential for lung metastasis. This phenomenon indicates that the tumour may evolve while growing along the postcava. To address this issue, we conducted multi-position sampling for each tumour (the distal end, the midpiece, and the proximal end) and then conducted RNA sequencing for each sample.

**Figure 2:**
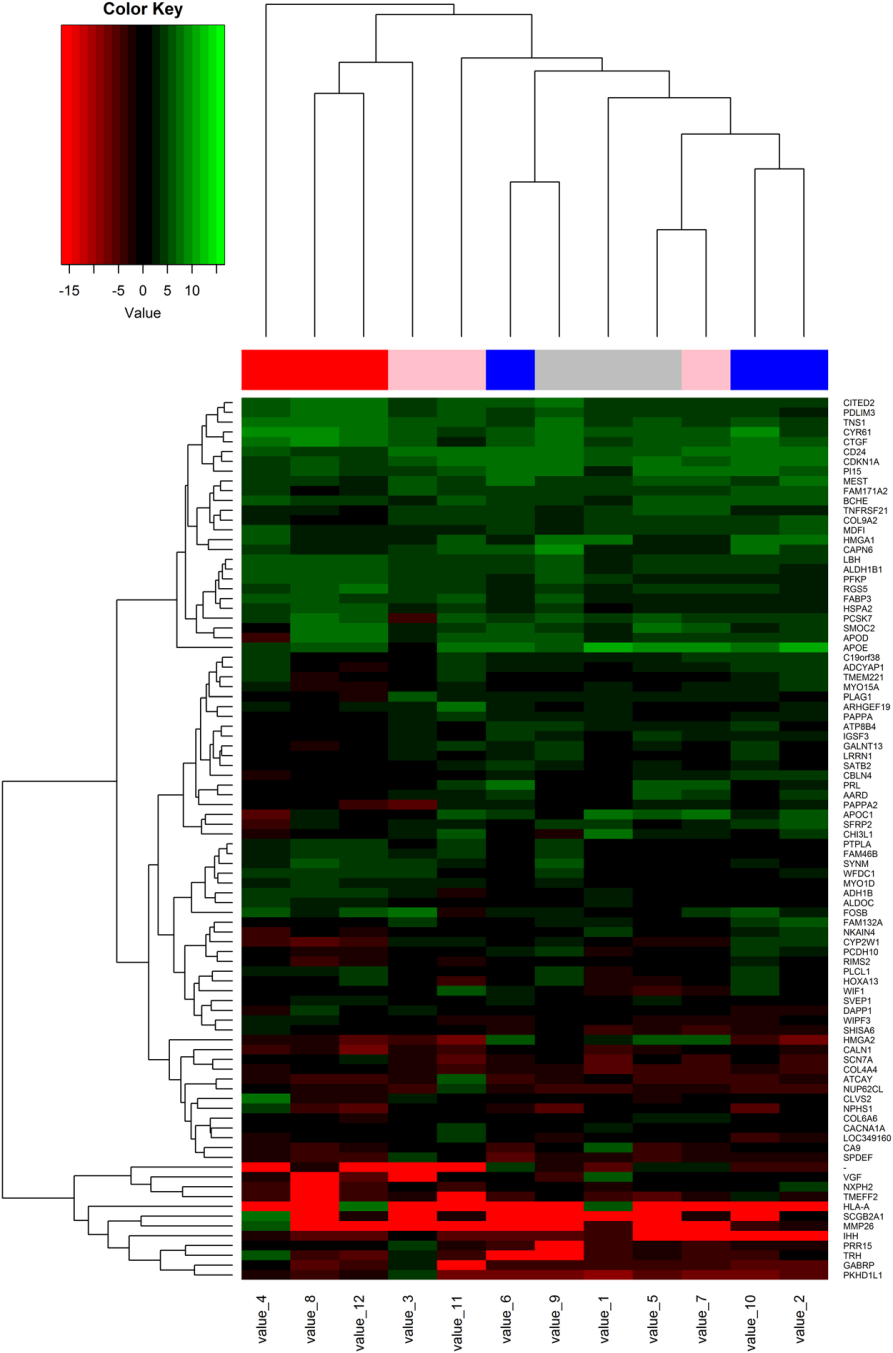
The heatmap was made by R software. The heatmap showed the DEGs expression of IVL specimes: beneath heatmap are the sequenced number representing specimen, On the right of heatmap are DEGs; On the left of the heatmap is colorkey.

**Figure 3:**
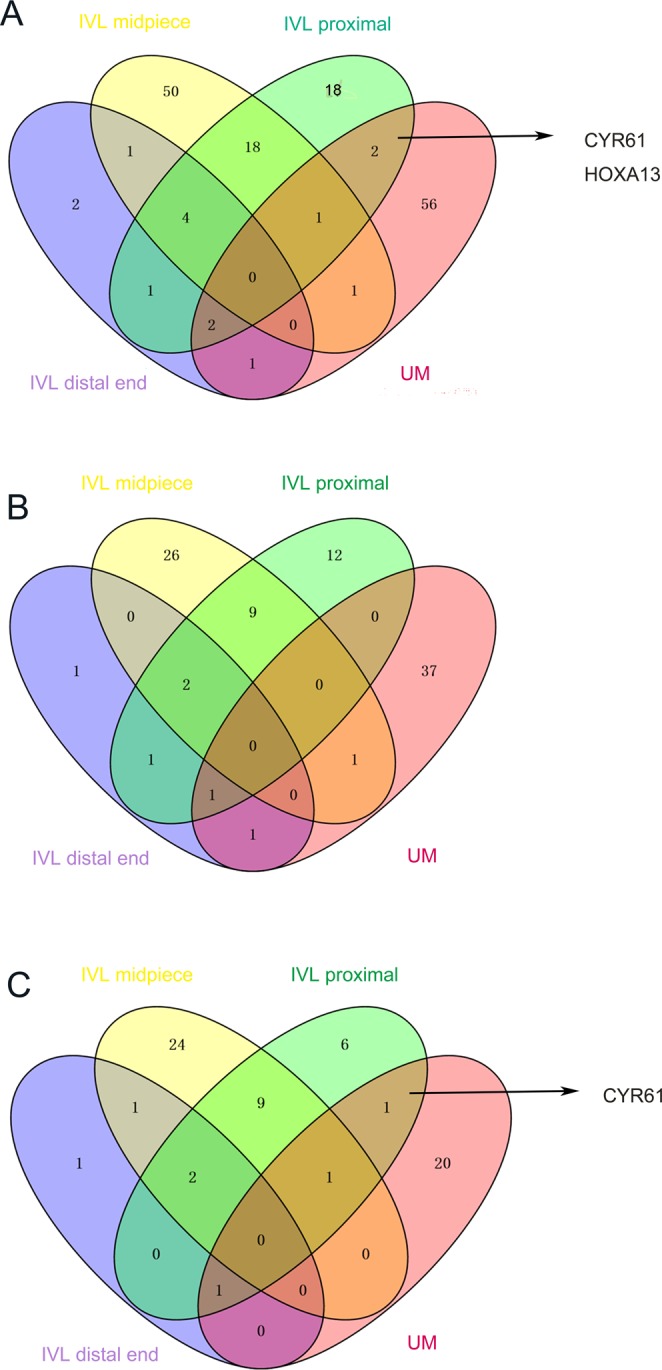
DEGs correlations of each IVL, UM and adjacent normal uterine muscle. (**A**) The IVL proximal shared two DEGs with UM: CYR 61 and HOXA 13 (**B**) The up-regulated DEGs correlations of each IVL, UM and adjacent normal uterine muscle. The IVL proximal shared no DEGs with UM (**C**) The down-regulated DEGs correlations of each IVL, UM and adjecent normal uterine muscle. The IVL proximal shared only one DEGs, CYR 61 with UM. It indicated that HOXA13 show a distinct dysregulation status in IVL and uterine myoma.

Compared with the paired myometrial tissues, we obtained 98 DEGs (Supplement materials) from the three parts of IVL (Fig. 2). Among the DEGs, 24.5% were shared between the proximal end and the midpiece of IVL, while only 5.1% were shared between the distal end and the midpiece (Fig. 3A). These results indicated the existence of extensive heterogeneous gene expression in IVL.

### Dysregulated gene networks in IVL

**Figure 4:**
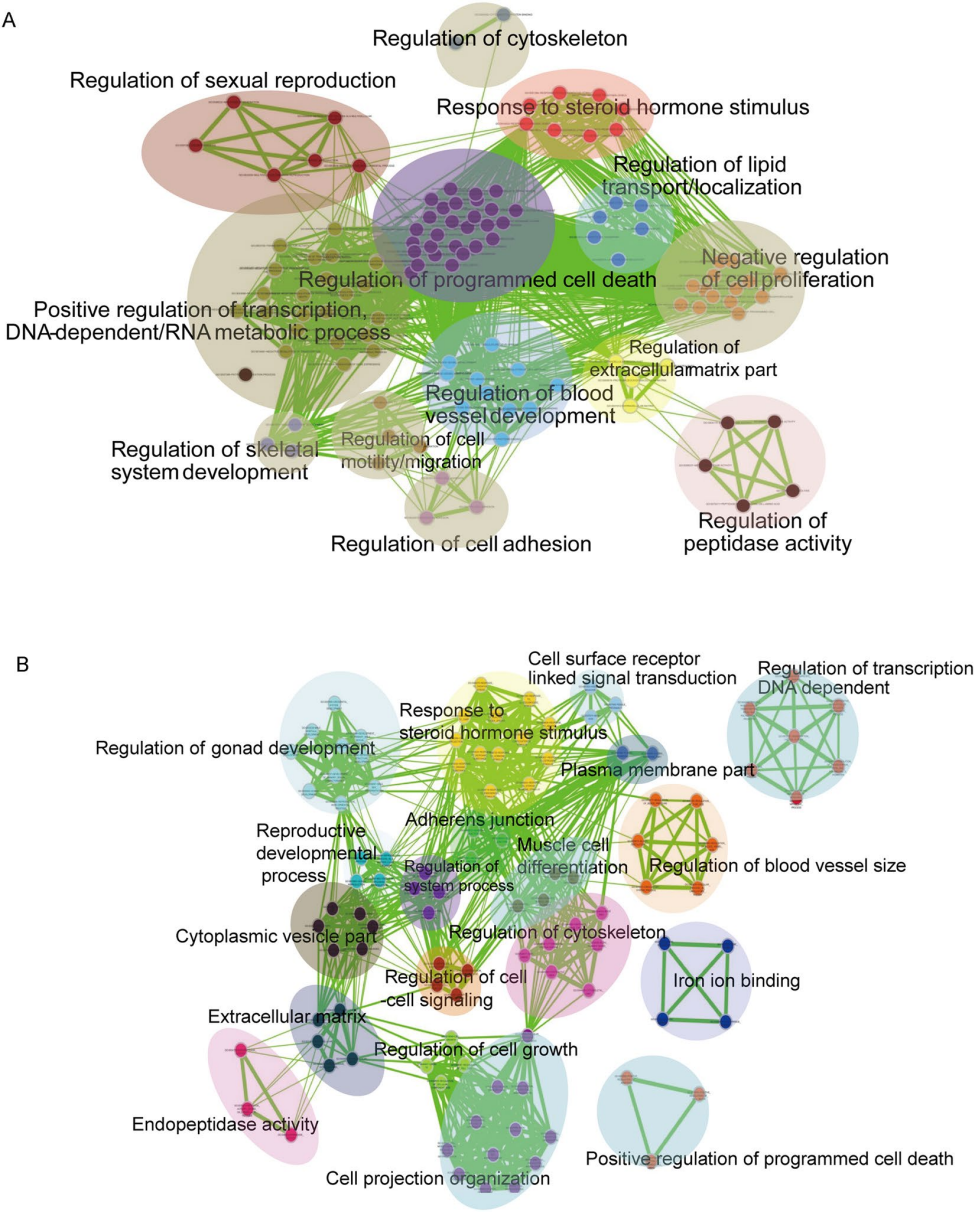
The gene sets for GO terms were visualized using the Cytoscape Enrichment Map plugin. Each node represents a GO term. Node size is indicative of the number of genes in a set. The thickness of each line is indicative of the number of genes shared between the connected gene sets. (**A**) The result showed DEGs enrichment in IVL. (**B**) Gene Ontology enrichment result of DEGs in uterine myoma.

The results of Gene Ontology analysis of the differentially expressed genes showed that the products of these genes were significantly associated with the extracellular region, acting in response to hormone stimulus, cell–cell adhesion, and in the regulation of cell growth and programmed cell death. Specifically, both the up- and down-regulated genes were particularly associated with response to hormone stimulus, extracellular matrix, cell adhesion, programmed cell death (Fig. 4A). This indicated that IVL may dysregulate the homeostasis of gene networks involved in hormone stimulus, the extracellular matrix, or programmed cell death.

### Dysregulated gene networks in uterine myoma

**Figure 5:**
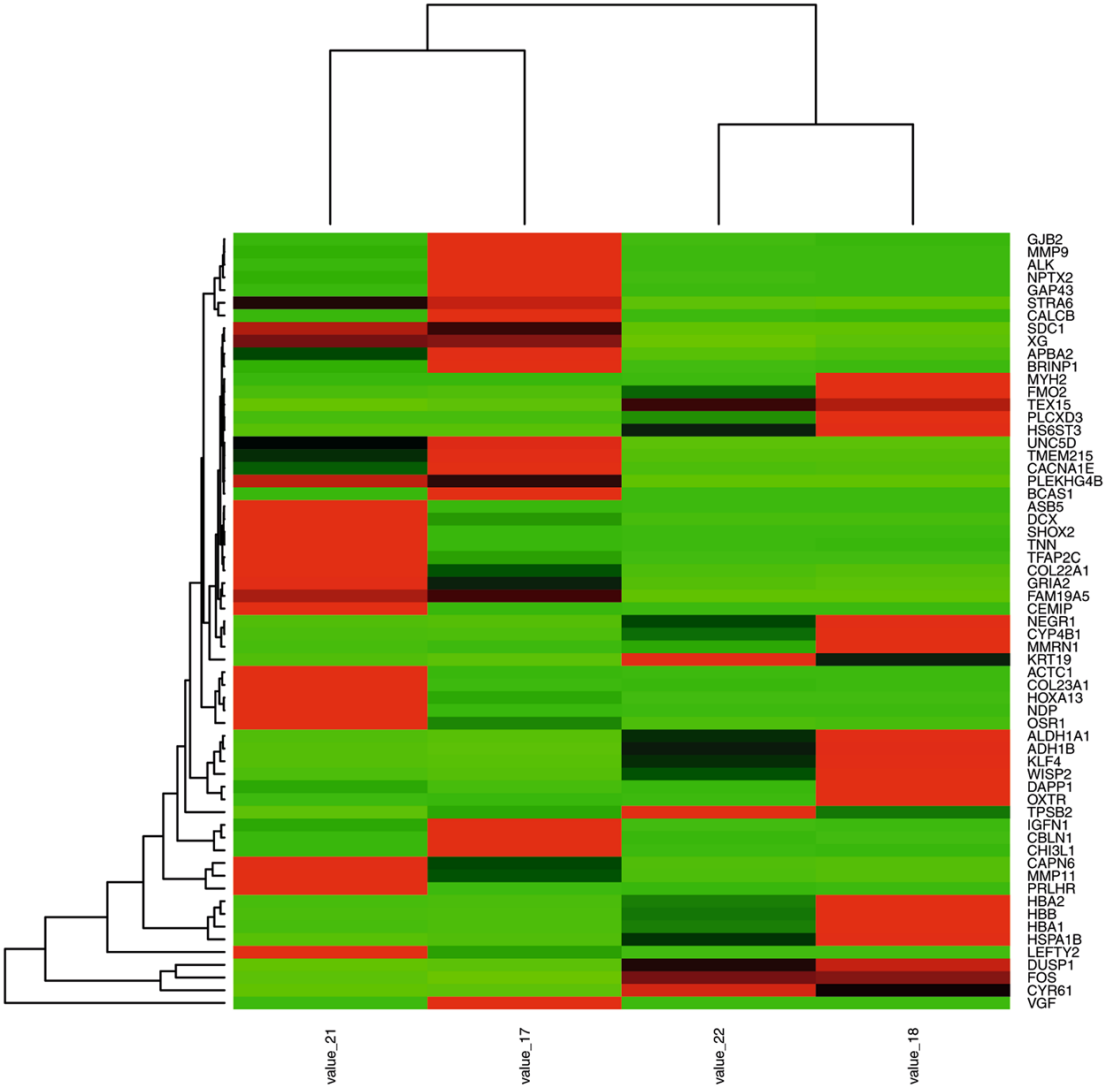
The DEGs heatmap of UM specimes: beneath heatmap are the sequenced number representing specimen, On the right of heatmap are DEGs; The colorkey is same with Fig. 2.

Owing to similarities in the clinical features of uterine myoma and IVL, we explored the dysregulated gene networks of uterine myoma. We conducted RNA sequencing for uterine myoma and paired myometrium, and obtained 61 DEGs (Fig. 5). Gene Ontology analysis results showed that these DEGs were particularly associated with response to steroid hormone stimulus, extracellular regions, and cell adhesion (Fig. 4B).

### IVL could be a subclass of uterine myoma

**Figure 6:**
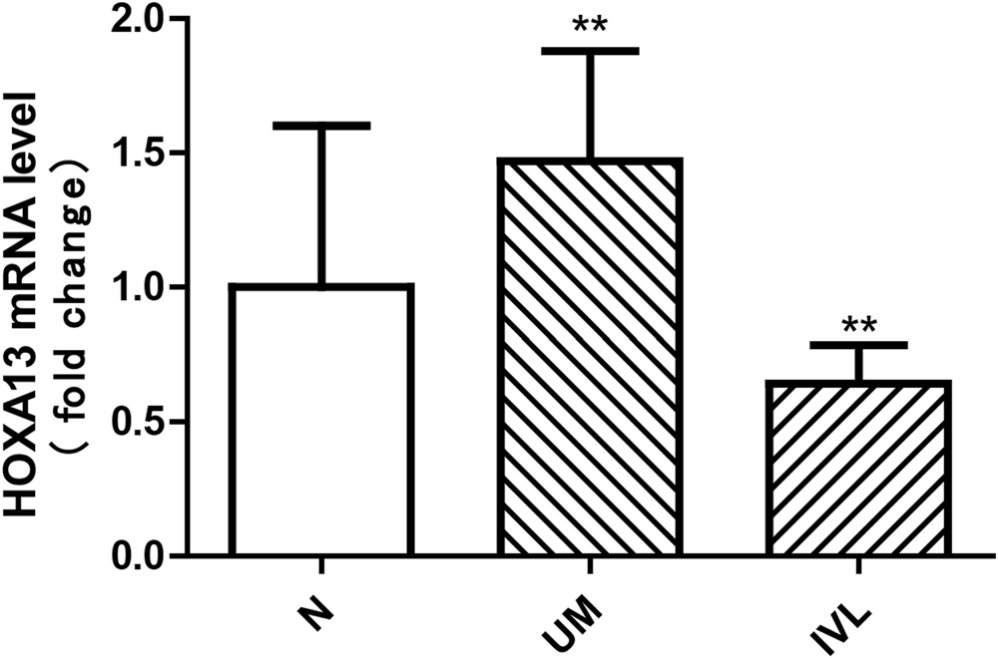
HOXA13 and GAPDH mRNA were detected by realtime polymerase chain reaction. P values were obtained by a one-way ANOVA. P < 0.05. The error bars were SEM. The fold change was 2. The result of HOXA13 RT-PCR showed that HOXA13 mRNA expression level in UM is higher compared with normal uterine muscle. On the contrary, HOXA13 mRNA expression level in IVL was lower than normal uterine muscle.

To further explore the relationship between IVL and uterine myoma, we compared the DEGs found in two diseases. As shown in Fig. 3A, only seven dysregulated genes were shared between IVL and uterine myoma and the IVL proximal end shared two DEGs with UM: CYR61 and HOXA13. When we took the dysregulation status into consideration, we found that HOXA13 is not included among the up-regulated DEGs (Fig. 3B) or down-regulated DEGs (Fig. 3C), showing that IVL and uterine myoma exhibited a difference in their dysregulation status. Next, we carried out real-time PCR of HOXA13 (Sangon Biotech; HOXA13 forward primer sequence: 5′-CAC GAA CCC TTG GGT CTT C-3′; HOXA13 reverse primer sequence: 5′-TCT TTG GGG CAG TAC ATT TGG-3′) to ascertain its expression level in IVL, UM, and normal uterine muscle (Fig. 6). The obtained findings revealed that HOXA13 may serve as a biomarker to distinguish IVL and uterine myoma.

We next explored the functional characteristics of the genes dysregulated in IVL and uterine myoma. We found that the differentially expressed genes of IVL and uterine myoma are both related to the dysregulation of steroid hormone stimulus, extracellular matrix, and cell adhesion. This indicated that IVL and uterine myoma might have similar dysregulated gene networks.

**Figure 7:**
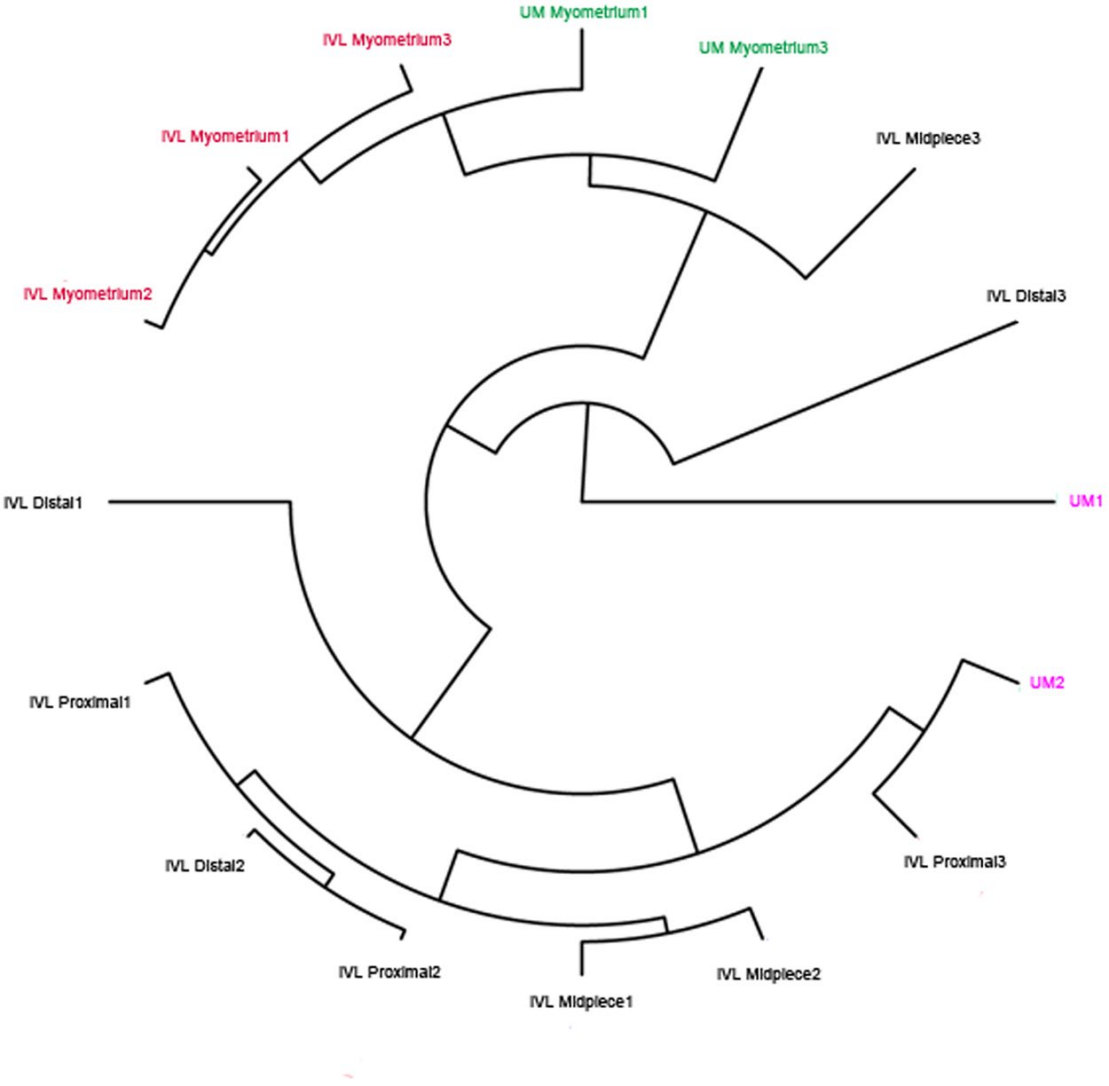
Cluster analysis was performed using the Cluster R package. The cluster analysis result showed that IVL and UM can not be seperated obviously in the dendrogram.

We extracted the expression profile of dysregulated genes in both IVL and uterine myoma and performed cluster analysis. As shown in Fig. 7, all myometrial samples were clustered together. However, IVL and UM could hardly be distinguished from each other. These results indicated that some IVLs are similar to UM.

## Discussion

IVL is a rare, benign, smooth muscle cell tumour. Few studies have been conducted to investigate its molecular changes or its relationship with uterine myoma. In this study, we employed RNA sequencing to explore the dysregulated gene networks in IVL and uterine myoma, to shed light on their relationship.

Since first IVL was identified, 200 cases have been reported in the literature^14^. IVL patients are typically middle-aged women, of whom approximately 50% have undergone a hysterectomy^15^. IVL is pathologically classified as a benign tumour, but behaves like a malignant lesion. IVL can extend into the atrium, leading to fatality^16^, or metastasize to the pulmonary artery^17^. However, with complete excision, the probability of recurrence is close to zero^15,18^.

The diagnosis and treatment strategy of IVL have been standardized, but its pathogenesis remains uncharacterized. Moreover, the origin of the tumour and the relationship between IVL and uterine myoma remains unknown. There are two main hypotheses about the origin of IVL. One suggests that it originates from the smooth muscle of the vessel wall, while the other suggests that it occurs due to uterine myoma extending through a vein^19^. For each of these theories, there are lots of supportive evidences.

One report of a pathological examination of the tumour revealed that it is benign and consists of proliferating smooth muscle fibres (rate of proliferation < 1%) with normal mitotic activity. Immunohistochemical analysis showed low levels of oestrogen receptor expression (around 30–40%), while progesterone receptor was strongly expressed (80%). These results suggest that IVL growth is connected to hormone levels^20^, which resembles the pathogenesis of uterine myoma.

Cytogenetic studies carried out to investigate IVL and uterine myoma suggested that spontaneous chromosome rearrangements may be responsible for the growth of uterine leiomyoma^21^. Leiomyosarcomas present complex karyotypic aberrations, while benign leiomyomas have some identified karyotypic abnormalities^22,23^. Bradley *et al*. studied the molecular cytogenetics of IVL, comparing it with uterine leiomyoma. They demonstrated that the pathogenesis of IVL is related to typical uterine leiomyoma and has a similar chromosomal mechanism. About half of uterine leiomyomas have structural chromosomal abnormalities, 20% of which show rearrangements of 12q14-15 targeting the gene encoding the high-mobility group AT-hook 2 (HMGA2)^24^. As this chromosomal rearrangement is also seen in IVL, some researchers think that uterine leiomyoma and IVL may represent different stages of a single disease. However, the uniclonal origin of IVL distinguishes it from multiclonal uterine leiomyoma^25^.

Another study used PCR sequencing to analyse nine IVL cases. The results demonstrated that all nine cases of IVL had a wild-type MED12 gene at codon 44, suggesting that IVL is a distinct smooth muscle tumour with a unique pathogenesis different from that of conventional leiomyomas. (The latter are associated with MED12 mutation.) Sekine *et al*. used Sanger sequencing to determine the prevalence of MED12 mutations in smooth muscle tumour of different organs. They found that, in contrast to uterine tumours, extrauterine smooth muscle tumours including leiomyomas, leiomyosarcomas, and angioleiomyomas do not have MED12 mutations^26–28^.

In summary, the relationship between IVL and uterine myoma has not been characterized, with no reported studies involving RNA sequencing. The current study may be the first to use RNA sequencing to investigate the origin of IVL. Our results showed that IVL and uterine myoma have similar dysregulated gene networks; both diseases are related to the dysregulation of steroid hormone stimulus, extracellular matrix, and cell adhesion. Moreover, clustering analysis showed that it is difficult to separate IVL from uterine myoma. These results indicated that IVL and uterine myoma might have similar origins. However, we found that only seven dysregulated genes were shared between IVL and uterine myoma. In addition, some genes playing a key role in regulating programmed cell death are significantly upregulated in IVL, but not in uterine myoma. Among these dysregulated genes, HOXA13 was shown to have a distinct dysregulated status between IVL and uterine myoma. These results indicated that IVL is not identical to conventional uterine myoma. In addition, HOXA13 may serve as a biomarker to distinguish IVL and uterine myoma.

## Conclusion

We concluded from this study that IVL is closely related to uterine myoma. While other study indicated that IVL cases may share some molecular cytogenetic characteristics with uterine leiomyoma^29^, IVL may be one subtype of uterine myoma, it is not identical to conventional uterine myoma. The HOXA13 gene showed a distinct dysregulated status between IVL and uterine myoma and may serves as a biomarker to distinguish these two diseases. The results of this study demonstrated that there is a close relationship between IVL and uterine myoma, but it still requires further exploration.

## Materials and Methods

### Patients and specimens

Fresh tumour samples and paired adjacent normal tissue were obtained from patients who underwent surgery at Peking Union Medical College Hospital between 2014 to 2016. Fresh samples were frozen with liquid nitrogen and stored at −80 °C. All patients were clinically diagnosed and the results were confirmed by two pathologists. IVL specimens were divided into three segments according to their morphology, namely, the proximal end, midpiece, and distal end, and then numbered sequentially (Table 2).

**Table 2:** The sequencing number of the IVL, UM and normal uterine muscle sample.

Patient	The distal	The midpiece	The proximal	Normal uterine muscle
IVL1	1	2	3	4
IVL2	5	6	7	8
IVL3	9	10	11	12
IVL4	13	14		
IVL5	15	16		
UM1			17	18
UM2			19	20
UM3			21	22

#### Statement

This study was approved by the Clinical Research Ethics Committee of Peking Union Medical College Hospital. Methods were carried out in accordance with the relevant guidelines and regulations. Written informed consent was obtained from each patient.

### RNA isolation and sequencing

We took 50 mg of tissue from each sample and used 1 ml Trizol (Invitrogen, Carlsbad, CA) to extract RNA from it, in line with the manufacturer’s instructions. The RNA of specimen numbers 13, 14, 15, 16, 19, and 20 failed the qualifying test. Qualified RNA was sequenced on the Illumina HiSeq™ 2000 platform (Illumina, San Diego, CA). Real-time PCR reactions were performed on the 7500 Real Time PCR System (Applied Biosystems, USA) to test the expression level of our target gene, HOXA13 (Sangon Biotech, Shanghai, China; HOXA13 forward primer sequence: 5′-CAC GAA CCC TTG GGT CTT C-3′; HOXA13 reverse primer sequence: 5′-TCT TTG GGG CAG TAC ATT TGG-3′). We used human GAPDH (Sangon Biotech, Shanghai, China) as an endogenous control. Experiments were performed in duplicate for each sample.

### Data analysis

Clean reads were mapped to human reference genome version 19 (hg19) using TopHat2^30^. Gene expression levels were calculated by Cufflinks-2.0.2^31^, and differentially expressed genes (P value < 0.05, q value < 0.05, fold change 2) were identified in accordance with the recommended procedure by Cuffdiff-2.0.2^32^. The heatmap of DEG was constructed using R software-3.4.3. We performed Gene Ontology enrichment analysis using The Database for Annotation, Visualization and Integrated Discovery (DAVID) version 6.7 (https://david-d.ncifcrf.gov/home.jsp)^33^ and the results were visualized using Cytoscape-2.8.0^34^. Cluster analysis was performed using the Cluster R package. RT-PCR data were analysed using GraphPad software. Results are expressed as mean ± SEM. A value of P < 0.05 was considered to be statistically significant. There were no non-default parameters were used for the analyses. Be aware that the software is updated on schedule, please use the latest version.

## Supplementary information

author information

dataset Gene functions

IVL gene data for heatmap

UM gene data for heatmap

## Data Availability

The datasets generated during and analysed during the current study are available in The European Genome-phenome Archive.The accession ID:EGAS00001002504.
