# Supplementary material for: Identification of the molecular relationship between intravenous leiomyomatosis and uterine myoma using RNA sequencing
Source: Sci Rep. 2019 Feb 5;9:1442. doi: 10.1038/s41598-018-37452-3 (PMC6363745; doi:10.1038/s41598-018-37452-3)
Supplement: Supplementary file 1 — author information [file 41598_2018_37452_MOESM1_ESM.docx]

**Title:**

Identification of the molecular relationship between intravenous leiomyomatosis and uterine myoma using RNA sequencing

**Author list**

Xu Zhang*, postgraduate student in PUMCH,e-mail:csurgeonzhangxu@sina.com

Liangcai Wu^*^, postgraduate student in PUMCH, e-mail: wsxpyzr@163.com

Rongjian xu*, MD student in PUMCH, e-mail: xrj3408@163.com

Chengpei Zhu, MD student in PUMCH, e-mail:pumchzcp@163.com

Guotao Ma, associate professor in PUMCH, e-mail:maguotao2000@163.com

Chaoji Zhang, associate professor in PUMCH, e-mail:chaojizhang@163.com

Xingrong Liu, associate professor in PUMCH, e-mail: liuxr@pumc.cn

Haitao Zhao, professor in PUMCH,email:zhaoHT@pumch.cn

Qi Miao, professor in PUMCH,email:miaoqipumc@hotmail.com

Postal address:

Department of Cardiac Surgery and Liver surgery,

Peking Union Medical College Hospital, Chinese Academy of Medical Sciences;

Beijing 100730, China

Telephone and fax:

Tel: 00861069152820 or Tel.: +86 10 69156042; Fax: 00861069152820 or Fax: +86 10 69156043.

Correspondence author email:

Correspondence author:

Qi Miao, email:miaoqipumc@hotmail.com

Co-Correspondence author:

Haitao Zhao, email: zhaoHT@pumch.cn

*Xu Zhang 、Liangcai Wu and Rongjian Xu contribute equally to the paper.

QiMiao is the correspondence author and Haitao Zhao is the co-correspondence author.
